# Cerebral Oximetry in Preterm Infants–To Use or Not to Use, That Is the Question

**DOI:** 10.3389/fped.2021.747660

**Published:** 2022-02-02

**Authors:** Gorm Greisen, Mathias Lühr Hansen, Marie Isabel Skov Rasmussen, Maria Vestager, Simon Hyttel-Sørensen, Gitte Holst Hahn

**Affiliations:** Department of Neonatology, Rigshospitalet and Department of Clinical Medicine, University of Copenhagen, Copenhagen, Denmark

**Keywords:** brain, oximetry, near-infrared spectroscopy, monitoring, randomized trial, clinical benefit

## Abstract

The Safeguarding the Brains of our smallest Children (SafeBoosC) project was initially established to test the patient-relevant benefits and harms of cerebral oximetry in extremely preterm infants in the setting of a randomized clinical trial. Extremely preterm infants constitute a small group of patients with a high risk of death or survival with brain injury and subsequent neurodevelopmental disability. Several cerebral oximeters are approved for clinical use, but the use of additional equipment may disturb and thereby possibly harm these vulnerable, immature patients. Thus, the mission statement of the consortium is “do not disturb—unless necessary.” There may also be more tangible risks such as skin breakdown, displacement of tubes and catheters due to more complicated nursing care, and mismanagement of cerebral oxygenation as a physiological variable. Other monitoring modalities have relevance for reducing the risk of hypoxic-ischemic brain injury occurring during acute illness and have found their place in routine clinical care without evidence from randomized clinical trials. In this manuscript, we discuss cerebral oximetry, pulse oximetry, non-invasive electric cardiometry, and invasive monitoring of blood pressure. We discuss the reliability of the measurements, the pathophysiological rationale behind the clinical use, the evidence of benefit and harms, and the costs. By examining similarities and differences, we aim to provide our perspective on the use or non-use of cerebral oximetry in newborn infants during intensive care.

## Introduction

Care of patients during acute illness has improved dramatically during the last 70 years (one point of departure was the polio epidemic in Copenhagen in 1951, where long-term ventilation was established for a large number of patients), and now routinely includes electronic continuous monitoring of vital functions and replacement therapy for organ dysfunction. It has typically been developed in a mechanistic, “plumber-like” manner, addressing one problem after the other within the “body-as-a-machine” paradigm. This is also true for newborn patients, although their small size, developing physiology as well as the fragility of their tissues pose special problems.

The adding of elements to the “life-support” package has been done over the years with variable levels of evidence. Marketing of medical devices, such as electronic patient monitors, is possible with documentation of safety and compliance with “essential performance.” For comparison, pharmaceutical products can only be marketed with proven benefits to patients. A central element in the process is the randomized controlled trial (RCT).

However, it takes an RCT with more than 1,000 patients to test if an intervention can reduce the risk of a complication from 10 to 5%. An even smaller reduction may be worthwhile, and it is hardly imaginable that all elements of life-support can be tested by RCTs. Each element can only be expected to contribute marginally to patient-relevant outcomes such as mortality, days alive outside hospital, motor or cognitive development, or the risk of neurodevelopmental disability.

Diagnostic or monitoring methods can only affect outcomes, if they lead to changes in management. Thus, a trial that tests the effect of a diagnostic tool on patient-relevant outcomes must have “thresholds of action” and be coupled to potentially effective interventions. Furthermore, when testing the value of a diagnostic method, it is the “added value” that matters. Typically, measures of pathophysiology may “overlap” as guidance for interventions, e.g., an abnormal value in one variable such as blood pH makes it more likely that another variable is abnormal, such as blood pressure, capillary refill or skin color. In a clinical trial, the experimental diagnostic method is typically tested in addition to “management as usual.” Finally, the patient-to-staff ratio and staff experience may influence the added value.

We will focus on four modes of continuous (time scale of seconds) electronic monitoring that all have as a purpose: To “safeguard the brain” by providing early warning of threats to its oxygen supply. Their clinical use differs markedly. Pulse oximetry is universally applied in neonatal intensive care and the use of invasive blood pressure monitoring is probably available in all neonatal intensive care units (NICUs) but used in an individualized way according to the precise clinical context. However, cerebral oximetry is used routinely in some NICUs and never in others, while electric cardiometry is not routinely used.

## Pathophysiology of Cerebral Hypoxia

Oxygen sufficiency of the brain depends on oxygen delivery, i.e., blood flow, blood oxygen content, and distances of diffusion from the capillaries to the mitochondria on one side, and oxygen consumption on the other. Blood flow is driven by pressure against vascular resistance, and blood hemoglobin concentration and the oxygen saturation of arterial blood determines the payload of oxygen. Blood pressure is generated by cardiac output against the total systemic vascular resistance.

Loss of function (electrical activity in the case of neurons) is the first consequence of acute oxygen insufficiency, and cellular injury occurs next. After an abrupt interruption of oxygen delivery, e.g., by cardiac arrest, injury starts within minutes, while it takes longer for injury to start when some oxygen is still delivered, e.g., in case of severe bradycardia, hypotension or hypoxia (1). The effects of longer-lasting subliminal oxygen sufficiency in terms of growth and development is less well determined.

Hypoxic-ischemic brain injury is relatively common in newborn infants, and it is most well-defined after a complicated birth, postnatal collapse, or neonatal stroke. It is now generally accepted that hypocapnic ischemia is a cause of white matter disease and cerebral palsy in preterm infants (2). Although low arterial blood pressure is associated with intraventricular hemorrhage, periventricular hemorrhagic infarction and death (3), not all authors agree that the epidemiological evidence is strong (4). Mechanistic reasoning indicates that the combination of marginal abnormalities in variables such as blood hemoglobin concentration, oxygen saturation, and blood pressure may add up to insufficient oxygen delivery.

The cardio-vascular transition after birth has specific aspects, including the potential for shunting through the arterial duct and the foramen ovale with increased pulmonary resistance. Filling of the heart may be compromised by positive airway pressure and the limited distensibility of the immature myocardium. The diving reflex is very active before and after birth, providing resilience during acute hypoxic stress to “vital” organs. While the brain is commonly counted as a vital organ, this is true for the brainstem, but may not be true for the hemispheres (5).

**Figure 1:**
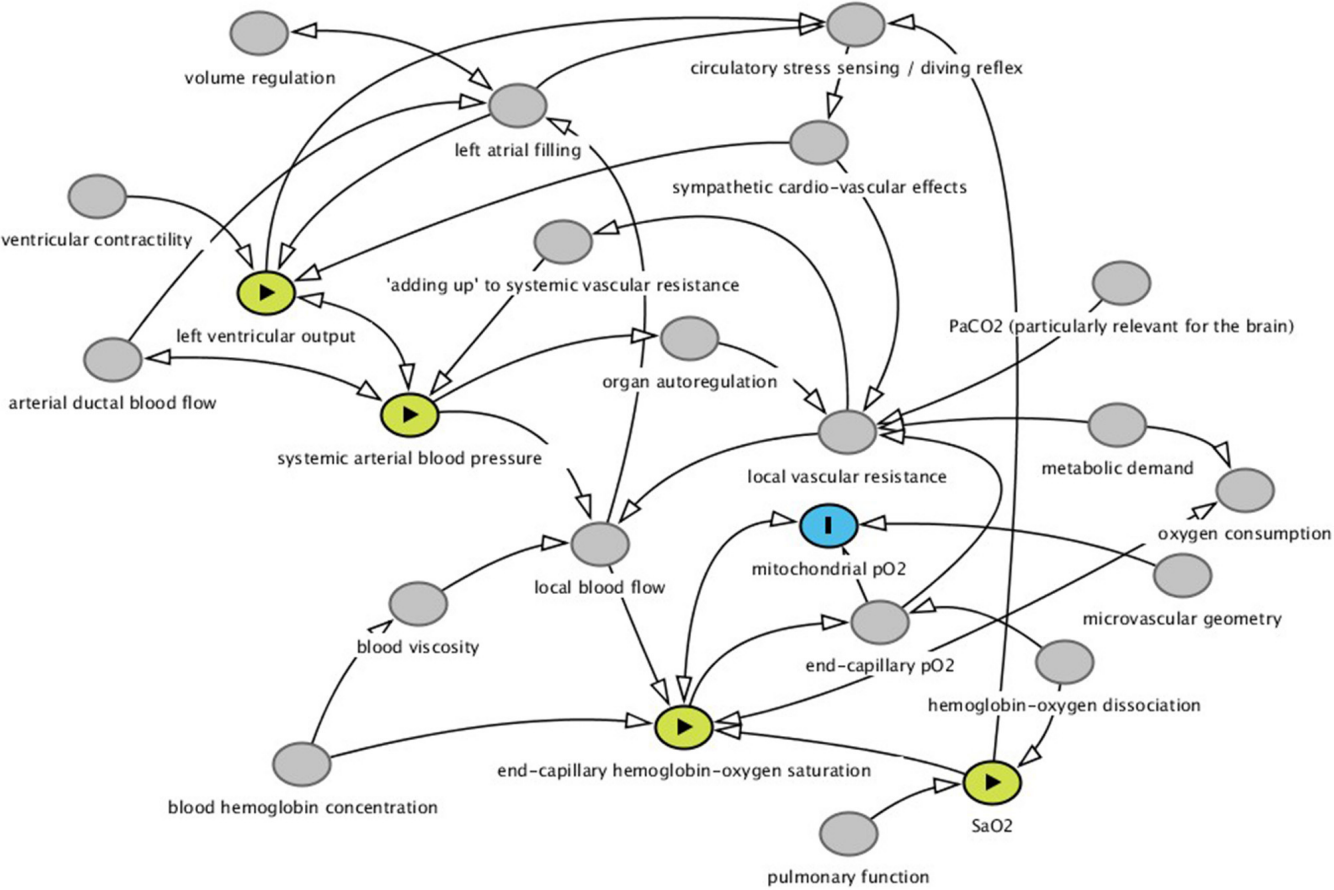
Simple mechanistic model of drivers of instant local tissue pO2 (colored in blue) i.e., the final common path to oxidative phosphorylation, oxygen consumption, local aerobic metabolism and organ function and health. The key concept in the model is that systemic vascular conductance (the inverse of resistance) is the sum of all local vascular conductances (in the figure it is labelled “adding up to systemic vascular resistance”). The figure is not an acyclical graph, since there is feedback at several levels. This makes questions of causation as well as the prediction of the effects of manipulation of variables more complex. Here we developed the model merely as an illustration of our view of the four physiological variables (labelled in yellow) for which the monitoring modalities discussed in this essay are more or less close surrogates. The figure was drawn in the Dagitty software for drawing causal graphs. The code can be found at http://dagitty.net/mQNlp4r and modified as needed for your purpose.

A simple model of neonatal circulation from a clinical perspective is illustrated in Figure 1. The four physiological variables, measured by the four monitoring modalities that we discuss here, are shown in color and their relation to the “mitochondrial oxygen tension,” which is the final point in the oxygen delivery machinery of the cardiovascular system. The arrows illustrate the direction of causation, but the fact that there is feedback at several levels indicates that it is not simple to predict the effects of a change in any single variable, even if we had access to much more information than we do.

## Cerebral Oximetry by Near-Infrared Spectroscopy (NIRS)

Cerebral near-infrared spectroscopy has been used in clinical research since the late 1970s. The method used to provide absolute values of hemoglobin-oxygen saturation as a percentage from 0 to 100%, appeared in the late 1990s. Cerebral oximetry was rapidly applied in the context of cardiac surgery and is used almost universally as clinical routine in the perioperative care of newborn infants with congenital heart disease and to some degree, also more generally in neonatology (6). Its mechanistic proximity to the final goal of safeguarding the oxygen delivery to the brain is compelling (Figure 1).

Randomized clinical trials have demonstrated that it is possible to reduce the “hypoxic burden” on the brain if the clinician has access to a real-time display of cerebral oxygenation when stabilizing very preterm infants in the delivery suite (7) or when providing cardio-respiratory support for extremely preterm infants during the first 72 h in the neonatal intensive care unit (8). A Cochrane review, however, concluded that the evidence for clinical benefit is very uncertain (9).

Tissue oximetry reflects the saturation in the circulating blood within the blood vessels according to their volume. It is therefore “venous-weighted,” and thus sensitive to the balance between local oxygen delivery and oxygen consumption. Tissue oximetry is not dependent on pulsation, and in the approved devices for clinical use, it is assumed that the tissues beneath the optode are optically homogenous. Inadequacy of this assumption may be the main reason for the limited repeatability, i.e., the standard deviation of repeated measurement after placing and replacing the sensor on the head of a newborn infant is typically around 5%. Recently, it was demonstrated that even the reactivity to arterial desaturations may vary considerably after replacement of the optode (10).

Only recently, an international standard has been issued (11) and due to the lack of a “gold-standard,” the readings of different devices or sensors may differ considerably (10, 12). Furthermore, the actual blood volume in the arterial, capillary and venous vascular compartments may differ among patients and change over time, so tissue oxygenation may change, even though end-capillary saturation stays constant.

Comparison of tissue oximetry to jugular venous oxygen saturation in pediatric patients showed limits of agreement of −12.5 to +10.1%, which is better than in adults (−20.7 to +19.7%) (13). However, jugular venous blood is not really a gold standard, since venous saturation is flow-weighted and therefore biased upwards by flow heterogeneity.

Finally, even end-capillary oxygen saturation does not accurately estimate mitochondrial oxygen tension due to variable diffusion distances, e.g., due to capillary loss and or tissue edema.

## Pulse Oximetry

The concept of pulse oximetry became clinically available in the early 1980s and became standard of care for anesthesia in The United States in 1986. Pulse oximetry is universally used in neonatology and depends on illumination of a significant arterial vascular bed and a measurable pulsatile signal. Co-oximetry on arterial blood provides a gold-standard for calibration and validation. Pulse oximeters approved for clinical use must have an accuracy (absolute root mean square) better than 3% in the range 70–100% as determined on healthy, adult volunteers. Unfortunately, accuracy may be less at oxygenation levels below 70%, and the agreement with co-oximetry was demonstrated to be surprisingly poor in a large study, using electronic patient data from clinical care in newborns (14).

## Electrical Cardiometry

Electrical cardiometry was developed in the early 2000s but has not gained any significant role in clinical care, perhaps partly due to the increasing availability of neonatologist-performed echocardiography or targeted neonatal echocardiography, the term used in North America.

Electrical cardiometry uses a weak high-frequency alternating current and four skin electrodes, to determine the pulsatile change in impedance or phase shift. The main signal source is assumed to come from the varying “alignment” of erythrocytes in the blood in the descending aorta, induced by the rapid changes in flow velocity—higher flow causes erythrocytes to align in columns. The signal is calibrated in the device and results are provided in common units as liters per minute and thus, can be directly compared to other measures. Limits of agreement when compared to echocardiography is in the range of ±30% (15). There is no validation in newborns against invasive measures of left ventricular output. The effect of a ductal shunt on the results depends on the direction of the shunt and may invalidate the comparison with left ventricular output, measured by other methods (16).

## Blood Pressure Measurement Through an Arterial Cannula

The first descriptions of monitoring the blood pressure through the umbilical artery in the hours after birth, appeared in the late 1960s. Invasive monitoring of arterial blood pressure is an established part of newborn intensive care, although clinical care guidelines may only offer broad advice to its use (17).

Arterial cannulas are also placed to ease blood sampling, partly for optimal blood gas measurement, and partly to reduce pain and stress when needing frequent blood sampling. Shortly after birth, umbilical artery catheters can be used. Recording blood pressure is technically simple and provided adequate zeroing, has few sources of errors. Blood pressure measurements derived from arterial catheters represent the gold-standard value, whereas the alternative, oscillometric, non-invasive measurement of blood pressure may not really be sufficiently reliable (18).

## Benefits, Harms, and Costs

Strong evidence of clinical benefit from randomized clinical trials is not available for any of the four modalities.

For cerebral oximetry, an attempt is currently in progress to obtain such evidence by a pragmatic randomized clinical trial, the SafeboosC-III trial (NCT03770741), enrolling 1,600 extremely preterm infants, to test the hypothesis that the risk of death or survival with severe brain injury can be reduced from 34 to 26.5% (19). Also, the COSGOD Phase III trial (Cerebral tissue oxygenation Saturation to Guide Oxygen Delivery, NCT 03166722) is underway, testing the benefit in the delivery suite (20).

Although pulse oximetry has been standard of care during anesthesia since 1986, a Cochrane meta-analysis including five trials that enrolled 22,000 patients during perioperative care, concluded that the evidence of clinical benefit was very weak (21). In neonatology, nearly 5,000 preterm infants have been randomized to five clinical trials, comparing a high and a low target range for arterial saturation as detected by pulse oximetry during the neonatal course (22). Although there was no difference to the combined outcome of death or major disability, the low target group had an increased risk of mortality, thus adding to the confidence that maintaining arterial saturation by pulse oximeter monitoring can help newborns to survive, although strictly speaking the low target may have been worse than no pulse oximetry at all.

The range of “normal” blood pressure has been widely discussed in the literature as the basis for treatment strategies. Ways to increase blood pressure have been widely studied in controlled trials. The clinical benefits, however, has mainly been studied using epidemiological methods, and only very recently a randomized, but unfortunately very underpowered, clinical trial was completed (23). As far as we know, little in this direction is underway for electric cadiometry.

The sensors used for cerebral and pulse oximetry may cause skin injuries due to a combination of heat and pressure. These sensors as well as the electrodes of electrical oximetry cause disturbance and may cause skin abrasions in the immature newborn, whereas the light and the electrical currents can be assumed to be without risks. The cannulas required for invasive blood pressure monitoring can cause thrombosis or embolism and result in loss of fingers, toes or part of extremities and in very rare cases, be life threatening.

In principle, for all four modalities there are risks involved in change of management induced by faulty readings or by the limited understanding of the pathophysiology involved. For instance, thresholds for action are not well established.

The costs of all four modalities are modest, at least when compared with the cost of some drug treatments, and when compared with the total cost of intensive care. Although there is an investment in monitors, and the sensors for cerebral oximetry are relatively expensive, the most important cost is probably the staff time involved in establishing and maintaining monitoring, as well as handling the results.

## Discussion of the Uptake of Medical Technology and the Potential for “Rolling it Back”

There are probably more similarities than differences between the four modalities. One hindrance to more widespread clinical uptake of cerebral oximetry in neonatology, may be the complexity of the physiology, doubts about the “added value,” and the sensor costs. In contrast, the main motive for introducing pulse oximetry may have been to protect against excessive oxygen therapy and the risks of retinopathy, and presumed oxygen toxicity in other organ systems. Furthermore, the direct response to changes in the fraction of inspired oxygen makes pulse oximetry intuitively useful.

Invasive blood pressure monitoring has been readily available in all neonatal intensive care units for many years. Still, it may be used less often now because of the concern that blood pressure is not really relevant, but blood flow is. Clinical use of electric cardiometry providing a measure of flow, however, is likely to have been held back by the limited reliability in terms of agreement with echocardiography, by the reservations about the meaning of cardiac *output in situations with variable* shunts through fetal channels, as well as the limited means of managing cardiac output. In some respects, monitoring of blood flow to relevant organs would be more appealing, but unfortunately, measuring cerebral blood flow with cerebral oximetry by NIRS appears to be even less precise than measuring tissue oxygenation (24).

On one side, as clinicians, we want to do the best for our patients. If a patient is critically ill and the balance of evidence is toward benefit, and our understanding is that it is mechanistically plausible that the patient should benefit, we may just use the technology to give them the best chance. This has been called compassionate use. We may end up implementing new interventions despite lack of robust statistical evidence. It may also provide comfort for parents and staff alike to know that “everything possible” has been done.

On the other side, as health service providers, we strive to deliver rational care based on evidence provided by RCTs, realizing the rising costs of medical care as well as the risks of medicalization, overdiagnosis, and overtreatment.

So, randomized clinical trials may have a place. The first question before planning a trial is if we have sufficient equipoise to inform parents and ask them for permission to let the child be randomized. It matters whether the trial will test something as a new option or something that has already been taken into routine use (25). Therefore, there is a case for doing a trial before adoption in clinical use, and one logical step, if in doubt about the clinical value, would be to take part in a trial.

There have been some examples of widely used intensive care treatments that have been “rolled back” after randomized trials after an intervention had achieved widespread implementation. The hydroxyethyl starch for volume expansion is one such example after an RCT showed that it increased, not decreased, mortality (26). In neonatology, mechanical ventilation was almost routinely used after birth in very preterm infants, before a series of RCTs demonstrated that prophylactic non-invasive respiratory support in combination with early rescue surfactant treatment would do better. Before that, RCTs had demonstrated that too much oxygen and indiscriminate use of postnatal steroids did more harm than good. In all these cases, treatments had gained widespread use, due to a mechanistic rationale and beneficial short-term effects.

The challenge of providing strong evidence is increased when it is extracted by meta-analysis from several RCTs, since it can be argued that repeated meta-analysis as well as heterogeneity among trials should be accounted for, e.g., by trial sequential analysis. This is fair, since a given intensive care element is likely to become an element in different life-support care packages. Patient populations are also likely to differ among hospitals, regions and countries. However, trial sequential analysis increases the required information size further and may make apparently conclusive metanalyses inconclusive—also those who point in the direction of harm (27).

## Conclusion: to use Cerebral Oximetry? or Not?

The clinical uptake of the four monitoring modalities differs widely and this is not based on clear differences as regards mechanistic rationale, nor evidence of clinical benefits. Rather, a variety of reasons may be in play.

Insufficient repeatability as well as unexplained differences among commercial devices is a limitation for cerebral oximetry. Only recently, an international standard has been published addressing the inability to extrapolate validation in healthy adult volunteers to sick patients or the anatomy of immature brains (11).

The SafeBoosC-III randomized trial testing the benefits and harms of cerebral oximetry during the first days after birth in extremely preterm infants will hopefully be published mid-2022 and will inform the decision as regards its use as clinical routine in this small, but high-risk group of patients.

Furthermore, it could be useful for other neonatal patient groups. The COSGOD-III trial tests the clinical benefit in the delivery suite. Preparations for a randomized trial in neonates in need of mechanical ventilation, SafeBoosC-IIIv are being made, with the goal of enrolling 3,000 newborns. The aim is to test the hypothesis that the risk of death or survival with moderate or severe neurodevelopmental disability at 2 years of age can be reduced from 20 to 16% by the addition of cerebral oximetry to the “intensive care package” that these children need. We invite others to join this effort (28).

More randomized trials will be useful, but questions will remain regardless of the results of these trials. Do the trials represent the conditions in my neonatal unit? If the trials are “negative,” are relevant effects really excluded?

Therefore, a better understanding of the causal pathways (Figure 1), better understanding of the effects of the interventions that are available during clinical care, and better definition of action thresholds are needed. So, if a decision is made to use cerebral oximetry as a clinical routine, then a good option would be to incorporate a systematic collection of data to describe the prognostic value of cerebral hypoxia and the responses to therapeutic interventions in carefully predefined formats and in numbers to enable firm statistical estimates. This last requirement calls for multicenter collaboration.

## Data Availability Statement

The original contributions presented in the study are included in the article/supplementary material, further inquiries can be directed to the corresponding authors.

## Author Contributions

GG drafted the first version. All authors discussed the substance, critically reviewed the draft, made changes, and approved the final version.

## Funding

Elsass foundation is the main sponsor of the SafeBoosC-III trial from where this perspective.

## Conflict of Interest

The authors declare that the research was conducted in the absence of any commercial or financial relationships that could be construed as a potential conflict of interest.

## Publisher's Note

All claims expressed in this article are solely those of the authors and do not necessarily represent those of their affiliated organizations, or those of the publisher, the editors and the reviewers. Any product that may be evaluated in this article, or claim that may be made by its manufacturer, is not guaranteed or endorsed by the publisher.
